# Monogenic Models: What Have the Single Gene Disorders Taught Us?

**DOI:** 10.1007/s11892-012-0325-0

**Published:** 2012-09-21

**Authors:** Tomasz Klupa, Jan Skupien, Maciej T. Malecki

**Affiliations:** 1 Department of Metabolic Diseases, Jagiellonian University, Medical College, 15 Kopernika Street, 31-501 Krakow, Poland; 2 University Hospital, Krakow, Poland; 3 Section on Genetics and Epidemiology, Joslin Diabetes Centre, Harvard Medical School, Boston, MA USA

**Keywords:** Monogenic diabetes, Gene, Mutation, Pharmacogenetics

## Abstract

Monogenic diabetes constitutes a heterogeneous group of single gene disorders. The molecular background and clinical picture of many of these diseases have been described. While each of these forms is much less prevalent than multifactorial type 1 and type 2 diabetes mellitus (T2DM), together they affect millions of patients worldwide. Genetic diagnosis, which has become widely available, is of great clinical importance for patients with single gene diabetes. It helps to fully understand the pathophysiology of the disease, tailor the optimal hypoglycemic treatment, and define the prognosis for the entire family. Monogenic diabetes forms can be divided into 2 large groups, resulting from impaired insulin secretion or from an abnormal response to insulin. There are several lessons we have been taught by single-gene diabetes. We learned that the gene responsible for the occurrence of diabetes can be identified if an appropriate search strategy is used. In addition, discoveries of genes responsible for monogenic disorders pointed to them as susceptibility candidates for T2DM. Moreover, establishing that some families of proteins or biological pathways, such as transcription factors or potassium channel subunits, are involved in monogenic diabetes sparked research on their involvement in multifactorial diabetes. Finally, the example of single gene diabetes, particularly *HNF1A* MODY and permanent neonatal diabetes associated with the *KCNJ11* and *ABCC8* genes, all efficiently controlled on sulfonylurea, inspires us to continue the efforts to tailor individual treatment for T2DM patients. In this review paper, we summarize the impact of single gene disease discoveries on diabetes research and clinical practice.

## Introduction

Physicians and researchers have been aware of diabetes mellitus heterogeneity for many decades. They well understood that hyperglycemia was just a common feature for a group of distinct disorders with different pathophysiology. First, they learned to distinguish the 2 most prevalent diabetic phenotypes–type 1 diabetes mellitus (T1DM), and type 2 diabetes mellitus (T2DM) [1, 2]. As soon as in the 1960s and 1970s, some experts realized that medicine was dealing with an even larger complexity of diabetes etiologies [3]. It was observed that in some families, the disease corresponded to neither insulin-dependent (T1DM), nor non-insulin-dependent (T2DM) diabetes. The affected individuals presented some features of both of the major diabetes forms, such as young age of onset and lack of obesity on the one hand, and clinical insulin independence on the other. This observation led to the description of the first monogenic forms of diabetes, maturity onset diabetes of the young (MODY) [4, 5]. Over the last decades more such monogenic forms of diabetes have been described [6–8]. It was also revealed that while each of these single gene diabetes forms were rare, they were numerous and, together, created a complex pathophysiological and clinical picture.

Beginning from the early 1990s, discovering the molecular background of monogenic disorders, including diabetes, became almost a routine procedure [9]. Thus, substantial progress was made in the dissection of the genetic background of monogenic forms of diabetes. The common denominators of these disorders are hyperglycemia and the occurrence of a severe single-gene mutation that causes the disease phenotype. Subsequently, it was possible to establish the specific diagnosis through the analysis of the patient’s DNA sequence. However, there are also substantial differences between these monogenic disorders. For example, based on pathophysiology, they may be divided into 2 major groups—associated with either beta cell defect or with severe insulin resistance (Table 1) [8, 10]. They also vary in respect of mode of inheritance, including autosomal dominant, autosomal recessive, and maternally inherited forms. Nevertheless, even in diseases with the same mode of inheritance interesting differences are observed, as in MODY families diabetes is passed from one generation to the other, while in permanent neonatal diabetes mellitus (PNDM) we observed many cases with spontaneous mutations [11]. These monogenic disorders are characterized by different ages of disease onset, from infants in PNDM, through adolescents in MODY, to middle aged subjects in some laminopathies [5, 8, 10, 11]. There are also substantial differences in their prevalence. For example, it is estimated that the proportion of MODY patients may constitute about 1 % of all diabetic cases, which would correspond to half a million patients on the European continent alone [12]. On the other hand, PNDM is very rare, it affects 1 child in 250 000 live births [11].

**Table 1:** Select important steps in unraveling genetic heterogeneity of monogenic diabetes

Beta cell disease		Insulin resistance	
Year	Disease	Year	Disease
1992–1999	HNF4A MODY, GCK, HNF1A MODY, HNF1B MODY, IPF MODY, Neurod1 MODY(formerly MODY 1-6)	1988	Type A insulin resistance
1992	MIDD	1988	Donohue syndrome
1998	Wolfram syndrome	2000	Familial partial lipodystrophy
2004–2006	Potassium channel sub-unit related PNDM	2002	Generalized lipodystrophy
2007	Insulin related PNDM		

Almost half a century of successful research constitutes an excellent perspective to look at what the single gene diabetes forms have taught us.

## Gene Search and Discovery

One important lesson that we learned from single gene diabetes is that the gene responsible for the occurrence of diabetes can be identified if appropriate methodology is used. The successful identification of monogenic diabetes genes encouraged and accelerated the genetic studies in T2DM. In general, there were 2 strategies used for the search for monogenic diabetes in the late 1980s and 1990s. The first one was the candidate gene approach, focused on a limited set of genes picked up for the mutation search based on their role in insulin secretion or insulin action. This strategy led to the discovery of the first monogenic diabetes genes—for example insulin receptor and glucokinase [13, 14]. It soon became clear that a more systematic strategy was necessary to increase the efficacy and success rate of the search for new genes. The strategy called genome-wide linkage study with subsequent positional cloning which led to the identification of the *HNF4A* and *HNF1A* genes, which were found to be responsible for 2 forms of monogenic diabetes, initially called MODY1 and MODY3, respectively [15, 16]. This discovery pointed to the entire family of transcription factors, leading to further single gene disorder discoveries, which involved *HNF1B, IPF, NEUROD1,* and some other genes [17–19].

The search for complex, common T2DM followed this general scheme seen in monogenic diseases. However, in polygenic diseases, scientists were looking for the associated susceptibility alleles with modest effect, rather than for sequence differences with strong causal effects. Although some sequence differences were identified as increasing the risk of T2DM using candidate gene association study design, for example the Pro12Ala variant of the *PPARG* gene, and the Glu23Lys of the *KCNJ11* gene [20, 21], this strategy brought many more disappointments than successes. Thus, as for monogenic diabetes, a more systematic strategy became desirable. Initially, researchers employed the same scheme of genome-wide linkage studies with subsequent positional cloning [22]. As, unlike in monogenic disorders, extended pedigrees were not available, large collections of small families were genotyped [22, 23]. Unfortunately, this strategy generated a significant amount of inconclusive data, unconfirmed findings, and, in general, was not successful. The only T2DM gene identified by this combined approach was *TCF7L2*, a transcription factor involved in the insulin secretion process [24]. The solution came together with high-throughput efficient genotyping methodology and a new generation of whole-genome search methods—genome wide association studies (GWAS) [25]. This systematic approach created a breakthrough and led to the identification of several dozens of genes whose variants confer small effect on T2DM susceptibility [26].

After the first set of single gene diabetes types was described in the 1990s, some researchers postulated that the possible involvement of these genes in common multifactorial forms of the disease should be tested. The hypothesis assumed that if a gene had been shown to carry a causal variant for monogenic diabetes with high penetrance, it may also include other variants with a detectable effect on T2DM. These variants could have an influence on gene expression or function with their impact being much too low to result in Mendelian disease inheritance but sufficient to modify the risk of a polygenic disease. Such small effect could be detected by case-control association studies. Not surprisingly, the initial studies were based on the candidate-gene strategy and involved genes identified as linked to MODY (*HNF1A, HNF4A*, glucokinase), PNDM (*KCNJ11, ABCC8*), and severe forms of insulin resistance (*PPARG*) [20, 27]. This phase of research was then followed by GWAS, which confirmed some of the earlier findings and added some new variants in monogenic diabetes genes associated with T2DM [26]. The list of genes responsible for monogenic forms of diabetes and associated with an increased risk of T2DM in GWAS is presented in Table 2.

**Table 2:** Variants within “monogenic diabetes” genes associated with T2DM—either identified or confirmed by large scale association studies

Locus	Chromosome	Monogenic phenotype	T2DM-associated variant
*PPARG*	3	Insulin resistance syndrome	Pro12Ala
*KCNJ11*	11	PNMD, TNDM, MODY	Glu23Lys
*ABCC8*	11	PNMD, TNDM, MODY	Ala369Ser
*HNF1B*	17	HNF1B MODY	rs3110641
*WFS1*	4	Wolfram syndrome	rs10010131, rs6446482
*HNF1A*	12	HNF1A MODY	rs7957197
*GCK*	7	GCK MODY, PNDM	rs1799884

GWAS identified many loci associated with the risk of T2DM and metabolic traits, such as obesity [26]. In spite of this substantial progress, the genetic loci so far identified are responsible for only a small portion of the overall genetic heritability of T2DM [28]. In addition, taken together, genetic risk markers have very limited application in individual risk prediction [29]. One of the new areas in the search is the identification of low-frequency, intermediate penetrance, moderate to large effect variants [30].

Whole genome sequencing or exome sequencing are novel approaches based on next generation sequencing [31]. They found application as a diagnostic tool, screening method for monogenic disorders and complex diseases [32]. Exome sequencing in diabetes and other common diseases reflects a paradigm shift in contemporary genetic studies: according to the novel hypothesis, T2DM and other polygenic disorders develop on a complex background of multiple rare variants with moderate to high impact on phenotype and variable penetrance, which obscures underlying Mendelian inheritance [33•]. This suggests that monogenic disorders might be pathogenetically closer to T2DM than it was previously perceived. Genes harboring mutations causative for monogenic diabetes are likely to contain low frequency causal variants in T2DM.

## Single Gene Diabetes Pathways and T2DM

Rare monogenic forms of diabetes with a single and well defined molecular cause help trace links between faulty genes, biochemical or regulatory dysfunction, and clinical phenotype. These forms can be considered simplified and partial models of T2DM, although, they cannot replicate the complexity of its pathogenesis. The process of insulin secretion is a particularly good illustration of how monogenic models help to understand the pathophysiology of T2DM. Glucose is transported inside pancreatic beta cells via several transporters and undergoes phosphorylation by the enzyme glucokinase, a specific form of hexokinase characterized by a low affinity to glucose and lack of inhibition by the product of catalyzed reaction, glucose-6-phosphate [34, 35]. The activated glucose enters glycolysis, the tricarboxylic acid cycle, and provides energy for adenosine triphosphate (ATP) synthesis in mitochondria through oxidative phosphorylation. The specific kinetic features of glucokinase, the gatekeeper of ATP synthesis process, make the amount of the ATP produced fairly proportional to the glucose concentration outside the beta cell. When the glucose level is low, the concentration of ATP remains low and ATP-sensitive potassium channels in the beta cell plasmatic membrane remain open. Potassium ion influx in the cell maintains the membrane potential. Increased glucose availability results in increased ATP concentration, closure of the ATP-gated channels, and activation of voltage-gated calcium channels. The influx of calcium in the cell signals for exocytosis of insulin granules [36].

Nearly every step of this process can be linked to a monogenic model of diabetes. Mutations affecting *GLUT2*, one of the glucose transporters in the human beta cells, result in the Fanconi-Bickel syndrome. This is a phenotypically complex glycogen storage disease with postprandial hyperglycemia or overt diabetes. Abnormal hepatic glucose clearance seems to be the major mechanism of hyperglycemia [37]. Impaired glucose sensing by beta cells also plays a significant role [38]. Recently, some very rare cases of PNDM have been linked to the mutations altering GLUT2 [39]. The finding that patients with homozygous mutations of the gene encoding GLUT2 develop neonatal diabetes suggests that this transporter plays an important role in the physiology of the beta cell. Loss of function mutations in *GCK*, a gene coding for glucokinase, result in a mild form of MODY [14]. The glucose sensing role of the affected gene product implies an intriguing phenotype: the blood glucose threshold for insulin secretion is increased, but the functional capacity of beta cells is preserved; a rightward shift of the dose-response curve is observed [40]. In addition, in a very rare biallelic inactivation of *GCK*, a phenotype of PNDM is observed: a complete loss of the capacity to respond to elevated glucose results in a phenotype of very early onset severe diabetes [41]. The next step in beta cell response to glucose, which is ATP synthesis, is impaired in a monogenic disease affecting mitochondria—maternally inherited diabetes with deafness (MIDD). MIDD results from the sequence differences of the mitochondrial genome and the severity of the diabetic phenotype may vary, from mild hyperglycemia to insulin dependence [42, 43]. The subsequent step of glucose sensing mechanism, the ATP-sensitive potassium channel consists of 2 subunits: channel-building Kir6.2, encoded by the *KCNJ11* gene, and regulatory SUR1, encoded by the *ABCC8* gene [44]. Activating mutations of either of these genes, keeping the channel in open conformation result in a diabetic phenotype, most commonly PNDM, less frequently MODY or relapsing diabetes [11, 44, 45]. Since *KCNJ11* is expressed outside the pancreas, some mutation carriers, in addition to insulin-dependent diabetes, present with neurological features such as developmental delay and muscle weakness [11, 44].

Beta cells may fail to produce and store a sufficient amount of fully functional hormone in their secretory granules. This happens when the *INS* gene coding for their content, insulin, is affected [46]. The defective and abnormally folded proinsulin molecule may induce the unfolded protein response and undergo degradation in the endoplasmic reticulum. It has been hypothesized that the resulting endoplasmic reticulum stress leads to beta cell apoptosis. The range of phenotypes varies from some mutations resulting in a complete destruction of beta cells and absolute lack of insulin and PNDM to others producing a MODY-like phenotype [47].

One of the most common causes of monogenic diabetes are sequence differences of genes encoding transcription factors. Their pathology is associated with impaired growth, differentiation and renewing of beta cells, as well as with an impaired transcription of the insulin gene. Transcription factor autosomal dominant mutations result in MODY. The genes that code for transcription factors associated with monogenic diabetes include *HNF1A, HBF1B, HNF4A, PDX1, NEUROD1,* and some others [15–19]. These transcription factors show varying expression patterns at different stages of pancreas development [48]. Selected genes and pathways of insulin secretion affected by monogenic diseases are shown in Table 3.

**Table 3:** Monogenic defects of insulin secretion

Genes	Function of the protein or tRNA	Phenotype of mutation carriers
*HNF1A, HNF1B, HNF4A, PDX1, NEUROD1, KLF11, PAX4*	Involvement in beta cell differentiation, proliferation and insulin synthesis	MODY, rarely PNDM if biallelic dysfunction
*BLK*	Non-receptor tyrosine-kinase regulating insulin synthesis	MODY
*SLC2A2 (GLUT2)*	Glucose transport to beta cell	Fanconi-Bickel syndrome
*GCK*	Glucose phosphorylation in the limiting step of ATP production in beta cell	MODY, PNDM if biallelic dysfunction
mitochondrial *leucyl tRNA* gene and several other mitochondrial genes	Mitochondrial ATP synthesis	MIDD
*KCNJ1, ABCC8*	Structure and function of the ATP-sensitive potassium channel of beta cells	PNDM, TNDM, MODY
*INS*	Coding for insulin	PNDM, MODY

Monogenic defects producing insulin resistance, the other fundamental component of the T2DM phenotype, seem to be less explored than single gene defects of insulin secretion. Nevertheless, a few examples of pathophysiological models are also available for severely impaired insulin action. Insulin receptor gene (*INSR*) mutations are linked to several syndromes of generalized insulin resistance. Their clinical presentation may be severe, with short stature, dysmorphic features, and short life expectancy, as in Donohue syndrome and Rabson-Mendenhall syndrome [49, 50]. These syndromes result from homozygous or compound heterozygous mutations [51, 52]. A milder form linked to the insulin receptor gene is type A insulin resistance, which may have dominant or recessive inheritance [53]. The patients may have less pronounced features present in pediatric syndromes, such as enlarged chin, nose, ears, and dental abnormalities. More importantly, phenotypic characteristics of INSR mutations include, in addition to hyperglycemia, other clinical features observed in T2DM and associated disorders, such as acanthosis nigricans, hirsutism, and polycystic ovaries. Mutations in the *AKT2* gene, coding for kinase, which plays a central role in post-receptor insulin signaling, produce insulin resistant diabetes and elevated fasting triglycerides, low HDL cholesterol, and high LDL level, a presentation similar to dyslipidemia accompanying T2DM. Patients also have liver steatosis, resembling the non-alcoholic fatty liver disease in T2DM [54]. *AKT2* mutation carriers also have reduced adipose tissue content. Congenital partial lipodystrophies resulting from mutations in *LMNA*, coding for nuclear envelope components, lamin A and C, or from mutations in gene coding the peroxisome proliferator activated receptor γ (*PPARG*) are characterized by abnormalities in the lipid profile (hypertriglyceridemia, low HDL-cholesterol), hirsutism, acanthosis nigricans, and hypertension [55, 56]. In these disorders we observe abnormal body fat distribution, which emphases the role of fat metabolism in the pathogenesis of insulin resistance.

In summary, it is quite clear that exploring monogenic diabetes provided new insights to a better understanding of the pathophysiological events leading to chronic hyperglycemia not only in single gene diabetes but also in complex forms of this disease.

## Pharmacogenetics in Diabetes

Another lesson coming from monogenic models of diabetes is that hypoglycemic treatment may be tailored for some patients based on the etiology of their diseases, which constitutes an example of pharmacogenetics. Pharmacogenetics is a scientific discipline that examines genetic variations that gives rise to differing response to drugs. This includes not only therapeutic efficacy but also toxic side effects of drugs.

Probably the earliest of pharmacogenetics is related to *HNF1A MODY* [7, 8, 16]. In this monogenic diabetes, pharmacologic treatment is usually inevitable; however, in spite of a frequently young age of diabetes onset, one can use a treatment alternative to insulin. Since sulfonylureas (SU), the oldest group of oral hypoglycemic agents, act on the beta cell and enhance insulin secretion [57], the notion that the latter group are the drugs of choice in MODY was very appealing. Several case reports from various populations suggested that patients with *HNF1A* MODY were characterized by a specifically prominent therapeutic response to SU [58, 59]. This hypothesis was eventually confirmed by a double blind, randomized, clinical study that compared the efficacy of gliclazide and metformin in *HNF1A* MODY and complex T2DM [60••]. *HNF1A* MODY patients showed much a better response to gliclazide than the patients with T2DM and this difference was associated with a great insulin secretion improvement in MODY patients. The effect of metformin on glucose levels in MODY patients was much weaker than for SU. The researchers attributed the findings to the nature of the beta cell defect in MODY, as *HNF1A* is responsible for the expression of genes involved in glucose uptake, glycolysis, and mitochondrial metabolism. SU act downstream to these cellular defects by binding to the *SUR1* subunit of the ATP-dependent potassium channel [57]. Thus, they are able to overcome the defects underlying this genetic form of diabetes and improve beta cell functioning. Thus, the genetic cause of *HNF1A* MODY is an important determinant of the response to oral hypoglycemic drugs. To date, the study of Pearson et al is the only randomized clinical trial performed in a monogenic population. The pathophysiological similarity suggests that SU are efficient in other MODY forms linked to transcription factors [7, 8, 15, 17–19, 61]. Unlike in transcription factor monogenic diabetes, in *GCK* MODY diet is sufficient in most affected subjects [7, 8, 62].

In some individuals affected by MIDD, the disease closely mimics T1DM and it requires insulin from the very diagnosis. In many other cases, secretagogues or even a diet alone are sufficient, at least for some time [63]. Metformin is generally considered contraindicated in MIDD patients because of the potential risk for lactate acidosis. However, this notion is based solely on the knowledge of the MIDD pathophysiology and biguanides mode of action rather than on clinical data.

Another spectacular example of tailoring hypoglycemic treatment to disease etiology is PNDM. Patients with *KCNJ11* mutations are characterized by a very good response to SU treatment. In this form of *KCNJ11*-related PNDM, SU action precisely corrects the mechanism underlying this type of diabetes by closing the activated potassium channel of the beta cells [11, 35, 64]. Successful transfer from insulin to SU has been accomplished for both adults and children, including infants [11, 65, 66, 67••, 68, 69]. In most of these cases, a substantial improvement in glycemic control was seen and no major side effects were reported. The doses of various SU compounds were much higher than usually therapeutically used in T2DM. It was hypothesized that SU use might be particularly useful in the correction of some extra-pancreatic symptoms, such as muscle weakness, or developmental delay. Indeed, recently published data provided evidence for neurological improvement in diabetic Kir6.2 mutation carriers after glibenclamide use [70]. In addition, most of the patients with a molecular diagnosis of PNDM due to *ABCC8* gene mutations could also be transferred off from insulin to SU [11, 47]. Unfortunately, we will not find such spectacular examples of pharmacogenetics for forms of diabetes associated with severe insulin resistance and clinical management of these patients remains challenging.

The important question that arises is whether we can translate the therapeutic success from some monogenic forms of diabetes to polygenic ones. Indeed, several common polymorphisms in genes linked to monogenic forms seem to influence the response to pharmacological treatment. The polymorphisms of *KCNJ11* and *TCF7L2* genes were associated with a therapeutic efficacy of SU in patients with T2DM [71, 72]. The *ABCC8* gene (encoding SUR1) Ser1369Ala variant was found to be associated with antidiabetic efficacy of gliclazide in the Asian population [73]. The Glu23Lys variant of *KCNJ11* was also associated with risk for severe SU-induced hypoglycemia in T2DM patients [74]. In a patch-clamp technique based study aimed to analyze nucleotide sensitivity and SU inhibition of recombinant human K_ATP_ channels containing *KCNJ11/ABCC8* variants, the increased sensitivity of the Glu23/Ala1369 variant combination to the SU-gliclazide was found [75]. However, realistically, we should not expect that in the nearest future pharmacological treatment in T2DM will be based on genetic testing. This stems from the fact that the response to treatment is probably influenced by many variants, each of them having little effect on the drug efficacy. It is possible that future discoveries of rare, major effect variants may add new perspectives for pharmacogenetics in T2DM.

## Conclusions

There are several lessons that we have learned from the monogenic diabetes research. The success in dissecting the molecular cause of rare diabetes forms encouraged and inspired genetic research of complex diabetes. The identification of new pathophysiological pathways for single gene diabetes enabled a better understanding of T2DM mechanisms. Spectacular pharmacogenetics examples in MODY and PNDM give us hope that, sometime in the future, hypoglycemic drugs will be tailored for other forms of diabetes based on their etiologies. Moreover, it seems that there is still some potential in exploring the role of genes in which mutations cause monogenic diabetes in polygenic forms of the disease. The application of novel technologies, including exome sequencing, may be useful. It may happen that the same new technology will be used for the search for novel mutations responsible for monogenic diabetes and “oligogenes” for common types of the disease. There is also a hope that new drugs targeting proteins and pathways altered in some forms of monogenic diabetes will be developed.

## References

[1] National Diabetes Data Group (1979). Classification and diagnosis of diabetes mellitus and other categories of glucose intolerance. Diabetes.

[2] Expert Committee on the Diagnosis and Classification of Diabetes Mellitus: Report of the expert committee on the diagnosis and classification of diabetes mellitus. Diabetes Care. 2003;(Suppl 1):5–20. 10.2337/diacare.26.2007.s5 12502614

[3] Tattersall RB, Fajans SS (1975). A difference between the inheritance of classical juvenile-onset and maturity-onset type diabetes of young people. Diabetes.

[4] Hattersley AT (1998). Maturity-onset diabetes of the young: clinical heterogeneity explained by genetic heterogeneity. Diabet Med.

[5] Fajans SS, Bell GI, Polonsky KS (2001). Molecular mechanisms and clinical pathophysiology of maturity-onset diabetes of the young. N Engl J Med.

[6] McCarthy MI, Hattersley AT (2001). Molecular diagnostics in monogenic and multifactorial forms of type 2 diabetes. Expert Rev Mol Diagn.

[7] Hattersley AT (2005). Molecular genetics goes to the diabetes clinic. Clin Med..

[8] Malecki MT, Mlynarski W, Skupien J (2008). Can geneticists help clinicians to understand and treat non-autoimmune diabetes?. Diabetes Res Clin Pract..

[9] Peltonen L, McKusick VA (2001). Genomics and medicine. Dissecting human disease in the postgenomic era. Science.

[10] Porter JR, Barrett TG (2005). Monogenic syndromes of abnormal glucose homeostasis: clinical review and relevance to the understanding of the pathology of insulin resistance and beta cell failure. J Med Genet..

[11] Rubio-Cabezas O, Klupa T, Malecki MT (2011). CEED3 Consortium Permanent neonatal diabetes mellitus–the importance of diabetes differential diagnosis in neonates and infants. Eur J Clin Invest..

[12] Malecki MT (2010). The search for undiagnosed MODY patients: what is the next step?. Diabetologia.

[13] Yoshimasa Y, Seino S, Whittaker J (1988). Insulin-resistant diabetes due to a point mutation that prevents insulin proreceptor processing. Science.

[14] Stoffel M, Patel P, Lo YM (1992). Missense glucokinase mutation in maturity-onset diabetes of the young and mutation screening in late-onset diabetes. Nat Genet.

[15] Yamagata K, Furuta H, Oda N (1996). Mutations in the hepatocyte nuclear factor-4alpha gene in maturity-onset diabetes of the young (MODY1). Nature.

[16] Yamagata K, Oda N, Kaisaki PJ (1996). Mutations in the hepatocyte nuclear factor-1alpha gene in maturity-onset diabetes of the young (MODY3). Nature.

[17] Horikawa Y, Iwasaki N, Hara M (1997). Mutation in hepatocyte nuclear factor-1 beta gene (TCF2) associated with MODY. Nat Genet.

[18] Stoffers DA, Ferrer J, Clarke WL (1997). Early-onset type-II diabetes mellitus (MODY4) linked to IPF1. Nat Genet.

[19] Malecki MT, Jhala US, Antonellis A (1999). Mutations in NEUROD1 are associated with the development of type 2 diabetes mellitus. Nat Genet.

[20] Altshuler D, Hirschhorn JN, Klannemark M (2000). The common PPARgamma Pro12Ala polymorphism is associated with decreased risk of type 2 diabetes. Nat Genet.

[21] Hani EH, Boutin P, Durand E (1998). Missense mutations in the pancreatic islet beta cell inwardly rectifying K + channel gene (KIR6.2/BIR): a meta-analysis suggests a role in the polygenic basis of Type II diabetes mellitus in Caucasians. Diabetologia.

[22] Botstein D, Risch N (2003). Discovering genotypes underlying human phenotypes: past successes for mendelian disease, future approaches for complex disease. Nat Genet.

[23] Turner RC, Hattersley AT, Shaw JT (1995). Type II diabetes: clinical aspects of molecular biological studies. Diabetes.

[24] Grant SF, Thorleifsson G, Reynisdottir I (2006). Variant of transcription factor 7-like 2 (TCF7L2) gene confers risk of type 2 diabetes. Nat Genet.

[25] McCarthy MI, Abecasis GR, Cardon LR (2008). Genome-wide association studies for complex traits: consensus, uncertainty and challenges. Nat Rev Genet.

[26] Visscher PM, Brown MA, McCarthy MI (2012). Five years of GWAS discovery. Am J Hum Genet.

[27] Vaxillaire M, Froguel P (2008). Monogenic diabetes in the young, pharmacogenetics and relevance to multifactorial forms of type 2 diabetes. Endocr Rev.

[28] Lango H, Palmer CN, Morris AD (2008). Assessing the combined impact of 18 common genetic variants of modest effect sizes on type 2 diabetes risk. Diabetes.

[29] Meigs JB, Shrader P, Sullivan LM (2008). Genotype score in addition to common risk factors for prediction of type 2 diabetes. N Engl J Med.

[30] Maher B (2008). Personal genomes: the case of the missing heritability. Nature.

[31] Ku CS, Cooper DN, Polychronakos C (2012). Exome sequencing: dual role as a discovery and diagnostic tool. Ann Neurol.

[32] Majewski J, Schwartzentruber J, Lalonde E (2011). What can exome sequencing do for you?. J Med Genet..

[33] Cirulli ET, Goldstein DB (2010). Uncovering the roles of rare variants in common disease through whole-genome sequencing. Nat Rev Genet.

[34] Matschinsky FM (1990). Glucokinase as glucose sensor and metabolic signal generator in pancreatic β-cell and hepatocytes. Diabetes.

[35] McCulloch LJ, van de Bunt M, Braun M (2011). GLUT2 (SLC2A2) is not the principal glucose transporter in human pancreatic beta cells: implications for understanding genetic association signals at this locus. Mol Genet Metab.

[36] Ashcroft FM, Rorsman P (2012). Diabetes mellitus and the β cell: the last ten years. Cell.

[37] Santer R, Schneppenheim R, Dombrowski A (1997). Mutations in GLUT2, the gene for the liver-type glucose transporter, in patients with Fanconi-Bickel syndrome. Nat Genet.

[38] Yoo HW, Shin YL, Seo EJ (2002). Identification of a novel mutation in the GLUT2 gene in a patient with Fanconi-Bickel syndrome presenting with neonatal diabetes mellitus and galactosaemia. Eur J Pediatr.

[39] Sansbury FH, Flanagan SE, Houghton JA (2012). SLC2A2 mutations can cause neonatal diabetes, suggesting GLUT2 may have a role in human insulin secretion. Diabetologia.

[40] Byrne MM, Sturis J, Clément K (1994). Insulin secretory abnormalities in subjects with hyperglycemia due to glucokinase mutations. J Clin Invest.

[41] Njølstad PR, Søvik O, Cuesta-Muñoz A (2001). Neonatal diabetes mellitus due to complete glucokinase deficiency. N Engl J Med.

[42] van den Ouweland JM, Lemkes HH, Ruitenbeek W (1992). Mutation in mitochondrial tRNA(Leu)(UUR) gene in a large pedigree with maternally transmitted type II diabetes mellitus and deafness. Nat Genet.

[43] Guillausseau PJ, Massin P, Dubois-LaForgue D (2001). Maternally inherited diabetes and deafness: a multicenter study. Ann Intern Med.

[44] McTaggart JS, Clark RH, Ashcroft FM (2010). The role of the KATP channel in glucose homeostasis in health and disease: more than meets the islet. J Physiol.

[45] Babenko AP, Polak M, Cavé H (2006). Activating mutations in the ABCC8 gene in neonatal diabetes mellitus. N Engl J Med.

[46] Støy J, Edghill EL, Flanagan SE (2007). Insulin gene mutations as a cause of permanent neonatal diabetes. Proc Natl Acad Sci U S A.

[47] Edghill EL, Flanagan SE, Patch AM (2008). Insulin mutation screening in 1044 patients with diabetes: mutations in the INS gene are a common cause of neonatal diabetes but a rare cause of diabetes diagnosed in childhood or adulthood. Diabetes.

[48] Servitja JM, Ferrer J (2004). Transcriptional networks controlling pancreatic development and beta cell function. Diabetologia.

[49] Donohue WL, Uchida I (1954). Leprechaunism: a euphemism for a rare familial disorder. J Pediatr.

[50] Rabson SM, Mendenhall EN (1956). Familial hypertrophy of pineal body, hyperplasia of adrenal cortex and diabetes mellitus; report of 3 cases. Am J Clin Pathol.

[51] Krook A, Brueton L, O’Rahilly S (1993). Homozygous nonsense mutation in the insulin receptor gene in infant with leprechaunism. Lancet.

[52] Kadowaki T, Kadowaki H, Accili D (1990). Substitution of lysine for asparagine at position 15 in the alpha-subunit of the human insulin receptor. A mutation that impairs transport of receptors to the cell surface and decreases the affinity of insulin binding. J Biol Chem.

[53] Odawara M, Kadowaki T, Yamamoto R (1989). Human diabetes associated with a mutation in the tyrosine kinase domain of the insulin receptor. Science.

[54] George S, Rochford JJ, Wolfrum C (2004). A family with severe insulin resistance and diabetes due to a mutation in AKT2. Science.

[55] Barroso I, Gurnell M, Crowley VE (1999). Dominant negative mutations in human PPARgamma associated with severe insulin resistance, diabetes mellitus and hypertension. Nature.

[56] Shackleton S, Lloyd DJ, Jackson SN (2000). LMNA, encoding lamin A/C, is mutated in partial lipodystrophy. Nat Genet.

[57] Gribble FM, Reimann F (2003). Sulphonylurea action revisited: the post-cloning era. Diabetologia.

[58] Sovik O, Njolstad P, Folling I (1998). Hyperexcitability to sulphonylurea in MODY3. Diabetologia.

[59] Pearson ER, Liddell WG, Shepherd M (2000). Sensitivity to sulphonylureas in patients with hepatocyte nuclear factor-1alpha gene mutations: evidence for pharmacogenetics in diabetes. Diabet Med.

[60] Pearson ER, Starkey BJ, Powell RJ (2003). Genetic cause of hyperglycaemia and response to treatment in diabetes. Lancet.

[61] Pearson ER, Pruhova S, Tack CJ (2005). Molecular genetics and phenotypic characteristics of MODY caused by hepatocyte nuclear factor 4alpha mutations in a large European collection. Diabetologia.

[62] Page RC, Hattersley AT, Levy JC (1995). Clinical characteristics of subjects with a missense mutation in glucokinase. Diabet Med.

[63] Maassen JA, T Hart LM, Van Essen E (2004). Mitochondrial diabetes: molecular mechanisms and clinical presentation. Diabetes.

[64] Sagen JV, Raeder H, Hathout E (2004). Permanent neonatal diabetes due to mutations in KCNJ11 encoding Kir6.2: patient characteristics and initial response to sulfonylurea therapy. Diabetes.

[65] Vaxillaire M, Populaire C, Busiah K (2004). Kir6.2 mutations are a common cause of permanent neonatal diabetes in a large cohort of French patients. Diabetes.

[66] Massa O, Iafusco D, D’Amato E (2005). KCNJ11 activating mutations in Italian patients with permanent neonatal diabetes. Hum Mutat.

[67] Pearson ER, Flechtner I, Njolstad PR (2006). Switching from insulin to oral sulfonylureas in patients with diabetes due to Kir6.2 mutations. Engl J Med..

[68] Tonini G, Bizzarri C, Bonfanti R (2006). Sulfonylurea treatment outweighs insulin therapy in short-term metabolic control of patients with permanent neonatal diabetes mellitus due to activating mutations of the KCNJ11 (KIR6.2) gene. Diabetologia.

[69] Malecki MT, Skupien J, Klupa T (2007). Transfer to sulphonylurea therapy in adult subjects with permanent neonatal diabetes due to KCNJ11-activating mutations: evidence for improvement in insulin sensitivity. Diabetes Care.

[70] Mlynarski W, Tarasov AI, Gach A (2007). Sulfonylurea improves CNS function in a case of intermediate DEND syndrome caused by a mutation in KCNJ11. Nat Clin Pract Neurol.

[71] Sesti G, Laratta E, Cardellini M (2006). The E23K variant of KCNJ11 encoding the pancreatic beta-cell adenosine 5’-triphosphate-sensitive potassium channel subunit Kir6.2 is associated with an increased risk of secondary failure to sulfonylurea in patients with type 2 diabetes. J Clin Endocrinol Metab.

[72] Pearson ER, Donnelly LA, Kimber C (2007). Variation in TCF7L2 influences therapeutic response to sulfonylureas: a GoDARTs study. Diabetes.

[73] Feng Y, Mao G, Ren X (2008). Ser1369Ala variant in sulfonylurea receptor gene ABCC8 is associated with antidiabetic efficacy of gliclazide in Chinese type 2 diabetic patients. Diabetes Care.

[74] Holstein A, Hahn M, Stumvoll M (2009). The E23K variant of KCNJ11 and the risk for severe sulfonylurea-induced hypoglycemia in patients with type 2 diabetes. Horm Metab Res.

[75] Hamming KS, Soliman D, Matemisz LC (2009). Coexpression of the type 2 diabetes susceptibility gene variants KCNJ11 E23K and ABCC8 S1369A alter the ATP and sulfonylurea sensitivities of the ATP-sensitive K(+) channel. Diabetes.

